# Techniques to Assess Long-Term Outcomes after Burn Injuries

**DOI:** 10.3390/ebj3020028

**Published:** 2022-04-20

**Authors:** Rae Spiwak, Shaan Sareen, Sarvesh Logsetty

**Affiliations:** 1 Department of Surgery, Max Rady College of Medicine, Rady Faculty of Health Sciences, University of Manitoba, Winnipeg, MB R3A 1R9, Canada; sarees41@gmail.com (S.S.); logsetty@umanitoba.ca (S.L.); 2 Department of Psychiatry, Max Rady College of Medicine, Rady Faculty of Health Sciences, University of Manitoba, Winnipeg, MB R3A 1R9, Canada; 3 Department of Children’s Health, Max Rady College of Medicine, Rady Faculty of Health Sciences, University of Manitoba, Winnipeg, MB R3A 1R9, Canada; 4 Manitoba Firefighters’ Burn Unit, Health Sciences Centre, Winnipeg, MB R3A 1R9, Canada

**Keywords:** burn, outcomes, administrative data, mental health, mixed methodology

## Abstract

Burn injuries have a tremendous impact on not only the physical health of the burn survivor, but also mental health and social outcomes of the individual and their support systems. While much effect occurs at the point of injury, post-injury pain, infection, scarring, inflammatory response and metabolic changes all impact the long-term health of the burn survivor. The goal of the following article is to explore how to examine long term outcomes associated with burn injury, including mental disorders, suicide, loss of work and quality of life in the context of risk factors for burn injury, including social determinants of health. We then discuss ways to examine post-burn outcomes, including the important role of administrative data, the advantages of mixed methodology research studies including qualitative research, and the importance of considering sex, gender and vulnerable populations, not only in study design, but in prevention and intervention programs.

## 1. Introduction

It is estimated that 11 million burn injuries occur annually worldwide, of which over 180,000 results in death [1]. Burn injuries have tremendous costs on both the individual and society. In 2018, it was estimated that the total cost of burn related injury was USD 299 million that year alone [2].

Burn injuries can be caused by multiple sources, including heat such as fire or hot liquids, cold, radiation, friction and electrical and chemical sources [3]. Regardless of the source, burn injuries and burn-related treatment can be traumatic to the burn survivor both physically and psychologically [4]. These injuries impact not only the burn survivor, but their families and support systems as well [5]. In addition to surgery, wound debridement and dressing changes can be painful, resulting in mental distress and disorders following acute injury [6,7]. While much damage occurs at the point of acute injury, post-injury pain, infection, scarring, inflammatory response and metabolic changes all impact the long-term health of the burn survivor. The goal of the following article is to highlight some of the risk factors for burn injury, including social determinants of health, as well as identify some of the long-term impact of burns, including mental disorders, suicide, loss of work and quality of life. Finally, we conclude with ways to examine these long-term outcomes, including the important role of administrative data, the advantages of mixed methodology research studies, including qualitative research, and the importance of considering sex, gender and vulnerable populations not only in study design but in prevention and intervention programs.

## 2. Risk Factors for Burn Injury

There are many factors that place some individuals at greater risk for burn injury and poor outcomes. Alcohol use and smoking, female sex, young age, violence, living in low- and middle-income countries, and individuals with lower socioeconomic status are all factors that increase an individual’s rate of burn injury and associated death [8]. Identification and understanding of these risk and social factors for burn injury are important as they can be used to develop effective burn prevention and intervention programs.

On a global scale, women have higher rates of deaths due to burn injury resulting from unsafe cooking environments (especially in low- and middle-income countries), and intimate partner violence (IPV) [9]. Violence against women and children is a significant cause of burns in all countries [8,9]. In a study by Spiwak et al., the prevalence of severe partner perpetrated burn among females in India was 1.00%, which is substantial given India’s large population (over 600 million females) [10]. This study also found that having an abusive father additionally increased the odds of severe partner perpetrated burn by over three times, when compared to participants that did not have an abusive father. Compared to women with IPV, greater wealth and rural status were protective against severe partner perpetrated burn. These findings are important as they signify the important and often overlooked role of social factors as they relate to burn injury. Swann et al., also highlight the importance of social factors in their investigation of burn injury and associated healthcare among Native American burn survivors [11]. The authors found Native American burn survivors were unique compared to non-Native American burn survivors with respect to average age, region, circumstances surrounding the burn, etiology of the burn, method of transport to the regional burn center, total length of stay, Injury Severity Score, total percent total body surface area burned, hospital charges, and payer’s source for medical costs. While differences between the populations differed, the authors argue that study findings largely resulted from the socioeconomic differences between the two populations [11].

## 3. Social Determinants of Health

**Figure 1:**
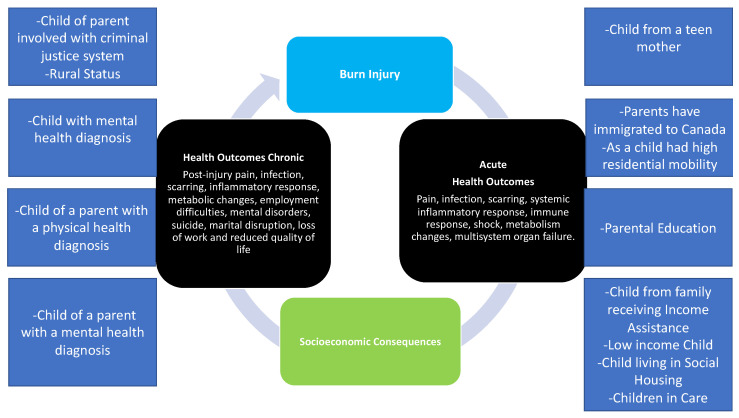
Conceptual causal model of social determinants of health, burn injury, and health outcomes. Adapted from Refs. [1,2].

An important and often overlooked category of predictors of burn outcomes are pre-existing social determinants of health (SDoH) that are related to the acute burn injury. SDoH are non-medical factors that impact health, such as income, housing, and childhood environment [3]. These factors influence health inequities and have been shown to impact health more than healthcare or lifestyle [3]. SDOH are an essential component of understanding burn injury risk and prevention (Figure 1). By examining burn-related injury and related measures of SDoH, we can generate evidence on risk factors for burn injury that informs interventions targeted early in the life course, possibly preventing the injury itself and modifying an individual’s future health and social outcomes. While some individuals are at greater risk for burn injury due to SDoH, burn injury itself can also impact an individual’s health and social status. For example, due to post-injury pain, an individual may have employment difficulties. As such, the burden of burn injury further marginalizes disadvantaged individuals as the burn injury itself acts as a determinant of future health [12].

Figure 1 presents a conceptual framework previously utilized by our team, examining 13 SDoH and its application to adult burn injury [12]. Our group has validated and successfully utilized these determinants in a study focused on pediatric burn injury [13,14,15]. Highlighting the powerful role of SDoH and burn injury, Padalko et al. found marked differences in SDoH between children that did and did not experience burn injury [16]. Specifically, children from low income households, children in care (children involved in the child welfare system), children from a family that received social assistance, and children born to teen mothers were at greater risk of burn injury [16]. Findings from this work are being used to develop burn prevention, education, and resources for young mothers who need support. While this framework highlights SDoH in the childhood/developmental period, the health impact of these factors reaches far into adulthood [17], including risk of burn injury. Future work should consider the powerful role of SDoH in the development of burn injury prevention programs for targeted populations.

## 4. Health Outcomes

### 4.1. Physical Health Outcomes

Given improved rates of survival and improvements in health care, survivors of burn injuries are living longer lives and more focus has been placed on post-injury care and consequences. Understanding the long-term consequences of burn injury is a vital component of the burn survivor’s health, with the long-term impact of burns, including physical disorders, mental disorders, suicide, marital disruption, loss of work and reduced quality of life. Physical conditions related to burn injury include hyper-inflammation, hypermetabolism, burn wound contractures, scarring and disfigurement, and chronic pruritus [18]. Seventy-five percent of individuals with burn injury experience chronic itching post-injury, with almost half of survivors experiencing pruritus more than four years later [19]. Other survivors experience burn-related pain for months and years following injury. In studies by Altier et al. (2002) and Gauffin et al. (2015), almost 30% of burn survivors had chronic pain for 63 months and 30% had pain for up to seven years post-burn (30%) [19,20]. While some survivors experience prolonged pain from their burn injury, others experience other symptoms such as fatigue and trouble sleeping [21]. All of these physical conditions can impact mobility, function and employment [22]. Scarring and contractures can impact the conduct of daily activities and mobility. The ability to perform daily activities and tasks is predictive of the burn survivor’s physical health related quality of life [22]. Returning to work is an important component of quality of life, and a third of burn survivors’ average return to work time is greater than two years post-injury [22]. In addition to pain and difficulties with mobility, other barriers to return to work include neurologic problems and mental health [22].

### 4.2. Mental Health Outcomes

With respect to mental health, the psychological consequences of burn injury can include depression [7], anxiety [7], and post-traumatic stress disorder (PTSD). Due to the physical conditions that can occur post-burn, including pain, scarring and disfigurement, as well as the traumatic nature of the acute burn, depression, anxiety, and PTSD are prevalent in a large proportion of cases. While there are factors that increase the likelihood of post-burn mental disorders, including being of female sex and having facial disfigurement, pre-burn related health, including depression, can increase an individual’s likelihood of experiencing post-burn mental disorders [23].

In a matched longitudinal cohort study, Logsetty et al. (2016) found that burn survivors had significantly higher rates of mental disorders including depression, and substance use disorder when compared to controls two years following burn injury [7]. However, these elevated rates were largely impacted by pre-existing mental disorders in the injured cohort. Other studies have also found elevated rates of pre-existing mental disorders among burn survivors [23,24,25,26], suggesting individuals with mental disorders may be at increased risk for burn injury. Another possible explanation may be that individuals with a history of mental disorders may be more likely to use alcohol and/or substances, which may increase their likelihood of injury overall [27]. Third, lower rates of post-burn mental disorders may have been found due to the extra care burn survivors may be receiving through support and follow-up care post injury. As a result, some of the potential negative mental health consequences may be mitigated in these populations. Finally, when using administrative data to examine health outcomes, methodological issues may play a role in the rates of mental disorders. Specifically, due to the fact physician diagnoses may be used to identify health outcomes, it may be a data coding issue where physicians bill for the primary visit (burn-related health concern) and not necessarily depression or anxiety, regardless of whether or not it was treated. As a result, mental disorders may have been underestimated in these types of studies. Although some studies have found that burns may not increase rates of mental disorders and health care utilization following injury, burn survivors are a vulnerable group who have a demonstrated increased rate of mental disorders and need for intervention. The studies mentioned highlight the importance of assessment and treatment of mood and anxiety disorders among burn survivors. In addition to mood and anxiety disorders, rates of alcohol and substance use disorders [7] have also been shown to be elevated in individuals with burn injuries [23]. Combined, these physical and mental health consequences can dramatically impact a burn survivor’s quality of life.

### 4.3. Quality of Life

Related to mental and physical health is health related quality of life (HRQOL). HRQOL is an outcome measure that reflects a burn survivor’s perception of their health on their physical, psychological and social wellbeing after a burn injury [28]. HRQOL has been increasingly studied among individuals with burn injuries [29]. Patients who have experienced severe burn injuries report lower quality of life scores, especially related to mental health (i.e., anxiety and depression) as well as pain [30,31]. HRQOL and mental health are closely related; in a review of 19 studies, Spronk et al., found burn severity, post-burn depression, post-traumatic stress, avoidance coping, reduced emotional or social support, and unemployment predict poorer HRQOL in burn survivors, highlighting the interrelationship between burn injury, mental health, and HRQOL [32].

Health-related quality of life (HRQoL) is an essential factor in treating people with burn injuries. However, striking the optimal balance between effective treatment and quality of life post-burn injury can be challenging. Health-related quality of life has increasingly been studied as various scales and surveys attempt to quantitatively or qualitatively assess HRQOL. Unfortunately, the singular best burn-specific scale or questionnaire is yet to be found, however, the Burn-Specific Health Scale–Brief (BSHS), Medical Outcomes Survey Short Form-36 (SF-36) and EuroQol (EQ-5D) questionnaires are often used [3]. Burn-specific surveys are being developed, however, the necessitate further validation, including the Brisbane Burn Scar Impact Profile, the CARe burn scale and the LIBRE survey [3,33,34,35,36].

A multitude of factors related to burn injury can affect a patient’s quality of life. Creating an all-encompassing assessment of the burn-injury quality of life proves to be a challenge due to the sheer amount of possible confounding variables. A review conducted by Stavrou and colleagues suggests that burns affect HRQoL in a multitude of ways, including physical appearance, relationships, and mental and physical health [37]. HRQoL scores are inconsistent and vary based on the healthcare facility and patient culture [36], with some studies finding that burn survivors experience a return to HRQoL levels comparable to the general population [38,39]. As most of such studies utilize generic measures such as the SF-36, burn specific measures are needed to determine if burn HRQoL is unique in requiring tailored tools to detect accurate measurements. A scoping review by Cimino et al., found that while the SF-36 is a widely used generic measure of HRQoL, has been validated in burn populations, and allows comparison to the general population, it does not consider issues that are specific to burn injury [40]. As such, they recommend that further studies may benefit from a combination of generic measures (i.e., SF-36) and burn-specific quality of life measures to obtain a better understanding of quality of life post-burn [40]. The current literature suggests that to provide the best treatment and quality of life for a burn patient, a clinician must apply tailored strategies and techniques depending on the patient’s individual circumstances.

## 5. Methods to Examine Long Term Outcomes

The study of outcomes following burn injury is methodologically challenging for several reasons including small sample sizes, selection bias, loss to follow-up, and difficulties with comparing burn survivors to other samples. Administrative data provides tremendous advantages in the study of burn-related outcomes, including the ability to: examine large populations without the limitations of recall bias, compare burn survivor populations to survivors of other forms of traumatic injury, study multiple factors using multilevel statistical models; examine data longitudinally and over the life course, and investigate other phenomena.

### 5.1. What Are Administrative Data?

Administrative data are comprised of information that is routinely collected from large populations for non-research purposes, and then merged at the individual level using de-identified data to be used in research [41]. In Canada, administrative data is collected for virtually all individuals due to universal health coverage, or a health care system that provides health care to all its citizens. As such, many populations can be studied, which is not always possible with survey data. Data are diverse and can originate from a variety of sources at multiple levels such as national, provincial and regional levels [42], and can contain information on health service utilization, education, social services or from registration offices such vital statistics [43].

Regardless of the type of administrative data, the use of linked databases enables researchers to investigate complex phenomena that may not be possible with individual or unlinked databases, including the study of rare events, longitudinal studies and inclusion of pre-existing factors such as income or health disorders.

### 5.2. Administrative Data Derived Health Indicators

Health indicators are measures that can represent a defined component of health including health status, non-medical determinants of health, health system performance community or health system characteristics [44,45]. Health indicators may also include a combination of factors, such as standardized health diagnoses for a grouping of individuals over a period of time [44]. Health indicators can be used to capture information from a variety of areas, which can then be used for many purposes including the assessment of health status for a population, the evaluation of health services received by a certain population, and the cross comparison of groups of individuals with different risk profiles [45]. The scope of health indicators can also be expanded to include risk and protective factors related to certain conditions or health outcomes [46].

For example, indicators can measure social determinants of health, in that non-medical characteristics such as region or household income can be used as social markers of risk or protection, or can be considered as a component of other health indicators [46], such as the case of some social indexes [41,47].

### 5.3. How Using Administrative Data Can Advance the Area of Burn Injury

#### Research

Administrative data derived health indicators could be used to determine whether burn survivors experience worse health outcomes as compared to other groups, including trauma survivors. Population-based administrative studies have not been widely used in burn care research [48], however the research that does exist originates mainly from Canada, Australia and Taiwan. These works have clearly demonstrated the advantages of population-based research for burn injury including the ability to follow populations over time, the ability to match burn survivors to other groups, examine pre and post burn health and social data, and also identify populations who may be at greater risk for burn injury, which is vital for evidence-based prevention [48]. Using administrative data, it is also possible to link many databases that may help explain why some individuals fair better or worse after burn injury. These databases can include hospital claims, medical claims, physician files, and vital statistics, as well as various other registries and social services data [49].

While access to such vast data provides researchers and policymakers with many investigational opportunities, it is necessary to use clearly defined, valid, and reliable health indicators. When using health indicators derived from administrative data, it is important to understand that data are collected for non-research purposes such as health care provider payment [41], therefore carefully choosing indicators is vital. Although some sources have cited the lack of data validation as a potential limitation when analyzing administrative data [50], the reliability and validity of some registries including the Manitoba Centre for Health Policy (MCHP) data registry have been examined and recognized [51,52,53]. These studies provide evidence for the use and validation of such measures related to the study of burn related outcomes, including measures of mood and anxiety disorder diagnoses [49].

### 5.4. Combining Data Sources to Study Burn-Related Outcomes

Administrative data can be augmented to improve validity, including the use of census and government data, and qualitative approaches including patient interviews, evaluation findings, and other data registries [54]. Although alternate sources of data can be used for augmentation, data may be of varying degrees of validity. For example, national health survey data may be limited in that data are subject to recall bias, individuals may not be aware that they had been diagnosed with a particular disorder, individuals may not report a mental disorder as they are asymptomatic following successful treatment, or individuals simply may not want to disclose personal health information [55]. For example, in a cross-validation study between a national health survey and administrative data, mental illness was underreported by the national survey by almost 50% [49]. These findings suggest that data obtained from medical charts or health care service billing may be more accurate at identifying an individual’s actual mental health state, which has significant implications for studying burn injury outcomes.

## 6. Strengths of Using Administrative Data Derived Indicators to Study the Consequences of Burn Injury

### 6.1. Population-Based Sampling

The use of health indicators derived from administrative data provide many general and specific advantages in the study of burn injury. Administrative data are rich sources of information, which can be used to answer questions without the methodological constraints present when conducting primary data collection or prospective trials. Specifically, population-based sampling is utilized, which ensures that data are representative of the entire population [56]. Self-selection, or selection bias, may result in the recruitment of individuals that may be functioning exceptionally well, therefore able to participate in research, whereas individuals who may be most in need of care and further exploration do not participate in the study. Similarly, the social stigma associated with mental health may prevent some individuals from participating in research, again resulting in a selection of cases that may be quite different from the population [57]. This selection bias may result in an incorrect portrayal of burn survivor outcomes, such as findings that underestimate poor health outcomes. While some previous studies of burn injury related outcomes have been limited by selection bias; the use of administrative data is not restrained by this limitation. As all individuals from a population contribute data, selection bias is addressed. As a result, representativeness is ensured, which is important for policy and health system approaches.

### 6.2. Longitudinal Analyses

The advantages of using administrative data to study long-term outcomes of burn injury allows the potential to study health outcomes over an extended time period (e.g., 10 years) and allows the analysis of a large sample of individuals. This ability increases the power of a study to find differences between injury cohorts if they exist. Also, a longer follow up period can be studied. As loss to follow up is an issue with longitudinal studies, it is a further advantage of utilizing administrative data as loss to follow up is greatly reduced. The use of population-based approaches to study longitudinal outcomes, such as a cohort study where burn survivors are followed up over a lengthy period of time, has demonstrated high external validity [7]. Secondly, recall bias is also eliminated. Health indicators such as physician diagnosed mental disorders or health care utilization are not limited by survivor recollection bias, as the study does not have to rely on an individual’s memory of health conditions. This factor is a key limitation in retrospective and longitudinal studies increasing the likelihood of data validity issues such as recall bias. Although the lack of recall bias is a potential strength of using administrative data, this strength also extends to the ability to identify family members of burn survivors to gain how the injury may have impacted them as well (i.e., parents, siblings, partners).

### 6.3. Ability to Study Multiple Factors Using Multilevel Statistical Models

The ability to examine the overlap in risk factors for poor health in burn survivors is another potential advantage of using administrative data to study burn injury. For example, individuals who have low levels of education or previous mental disorders prior to burn injury may be placed at different risk for poor health following injury independent of the burn injury itself [3,5,7,58]. Health indicators derived from linked data enable the examination of these potentially overlapping factors [41], which can then be analyzed using complex statistical models [49].

### 6.4. Capture Marginalized Populations

An additional strength of using administrative data to study burn outcomes is that it allows investigation into underrepresented groups. Data from burn survivors could also be aggregated or broken down at different geographic or administrative levels enabling the analysis of individuals belonging to different communities. Including factors such as sex, region, and income in analyses, can be used to ensure populations of study are comparable by incorporating various population characteristics that may be related to burn outcomes, as well as help determine differences in population health outcomes [47].

### 6.5. Ascertainment of Mental and Physical Disorders

The use of derived health indicators for both burn survivors and their support systems has many advantages, such as the ability to assess both pre and post injury health disorders [49].

Unfortunately, measures of mental and physical disorders only represent individuals who sought or received care from a care provider or who were hospitalized for a related event [40]. These health indicators, while physician diagnosed, report treatment or use prevalence, which limit generalization to individuals who did not seek care [59,60]. It has been shown that failure and delays in help seeking for mental disorders is a problem worldwide [61], with older individuals, men, and individuals in developing countries being less likely to seek care, potentially making them appear to have fewer outcomes of interest [62]. Barriers to help seeking include desires for an individual to manage the problem on their own, lack of awareness or knowledge about where to find care, and unavailability of professional help [63]. As a result, measures of mental disorders are likely underestimates of actual disorders. Although many disease diagnoses are available using administrative data, some important non-diagnostic factors such as PTSD and suicide ideation are not available. For example, in Manitoba some disorders such as posttraumatic stress disorder (PTSD) diagnoses are not available due to limitations in coding diagnoses. PTSD cannot be differentiated from other anxiety conditions in ICD coding, as some jurisdictions do not code all decimal places. Although these factors may be difficult to assess using administrative data, they may be possible to examine using alternate data sources, such as the inclusion of suicidal ideation through examination of Canadian Community Health Survey data [64].

## 7. Social Factors

Social measures including information on social supports, including close friendships and relationships, as well as emotional components of burn injury and patient culture are not commonly available in the administrative data, representing a significant limitation in the study of long-term outcomes.

Social supports and community integration are important predictors of post-burn injury functionality, and furthermore, psychosocial outcomes are an important component of an individual’s experience and response to burn injury [65,66,67]. Research has shown that more social supports are related to positive outcomes. Additionally, factors including quality of life, work satisfaction, and relationship quality and characteristics are important social factors that impact an individual’s experience and adjustment to burn injury [68]. Stigma is another important consideration in the study of mental health outcomes from injury as it can act as a barrier to treatment or help-seeking [69]. Due to the limitations of using administrative data and the importance of investigating the role that social factors play in outcomes following burn injury, it is important to pursue other methodologies that can address these areas.

## 8. The Importance of Qualitative Research and Mixed Methodology in the Study of Burn Outcomes

While administrative data provides many advantages for understanding the long-term outcomes associated with burn injury, qualitative research is an important research approach that can provide a more complete picture of a burn survivor’s experience of injury, as well as post-injury adjustment. Recent work has emphasized the importance of qualitative research and mixed methodology in understanding burn injury and care due to its ability to describe the lived experiences of burn survivors and care providers [70,71]. While qualitative methodology consists of many approaches, such as phenomenology, ethnography and grounded theory, the strengths are the ability to examine sensitive topics, recognition of subjectivity and bias, the importance of participant and researcher histories and assumptions, and the ability to analyze information robustly. Qualitative methodology can also be combined with quantitative methods to produce a mixed methods study, where both qualitative and quantitative methods are used to provide a comprehensive analysis of a research question or area. While qualitative findings reflect the sample investigated and are not generalizable, this methodology can delve into social factors such as stigma, feelings of guilt and blame, body image, loss of relationships, need for psychosocial support and areas that public health and policy makers can utilize to create intervention and prevention programs, such as the importance of providing transportation options and psychosocial follow-up for burn survivors [71,72].

## 9. Conclusions and Future Directions

Investigation into the health and social outcomes of burn survivors is an important and emerging field. While much work has been done, future work on social determinants of health, including particular focus on at-risk groups, sex differences in outcomes, and understanding social constructs, such as how gender may impact risk for burn injury and outcomes post-burn, would be a tremendous addition to this important area. Furthermore, long-term examination of burn outcomes (greater than five years post-burn), is needed to understand the impact of burn injury over time [40]. Finally, novel strategies to examine HRQoL that include a combination of both generic health and burn-specific HRQoL are needed to truly understand and accurately measure quality of life among burn survivors [40].

## Data Availability

Not applicable.
